# Age-dependent cerebral vasodilation induced by volatile anesthetics is mediated by NG2^+^ vascular mural cells

**DOI:** 10.1038/s42003-024-07200-7

**Published:** 2024-11-15

**Authors:** Hang Zhou, Viola Neudecker, Jose F. Perez-Zoghbi, Ansgar M. Brambrink, Guang Yang

**Affiliations:** 1 Department of Anesthesiology, Columbia University Irving Medical Center, New York, NY 100032 USA; 2 Present Address: Faculty of Life and Health Sciences, Shenzhen University of Advanced Technology (SUAT), Shenzhen, Guangdong Province 518107 China

**Keywords:** Neuroscience, Circulation

## Abstract

Anesthesia can influence cerebral blood flow by altering vessel diameter. Using in vivo two-photon imaging, we examined the effects of volatile anesthetics, sevoflurane and isoflurane, on vessel diameter in young and adult mice. Our results show that these anesthetics induce robust dilation of cortical arterioles and arteriole-proximate capillaries in adult mice, with milder effects in juveniles and no dilation in infants. This anesthesia-induced vasodilation correlates with decreased cytosolic Ca^2+^ levels in NG2^+^ vascular mural cells. Optogenetic manipulation of these cells bidirectionally regulates vessel diameter, and their ablation abolishes the vasodilatory response to anesthetics. In immature brains, NG2^+^ mural cells are fewer in number and express lower levels of Kir6.1, a subunit of ATP-sensitive potassium channels. This likely contributes to the age-dependent differences in vasodilation, as Kir6.1 activation promotes, while its inhibition reduces, anesthesia-induced vasodilation. These findings highlight the essential role of NG2^+^ mural cells in mediating anesthesia-induced cerebral vasodilation.

## Abstract (web summary)

Live animal imaging reveals age-dependent cerebral vasodilatory responses to volatile anesthetics, pronounced in adult mice and diminished or absent in developing brains. These effects are mediated by NG2^+^ mural cells and Kir6.1 signaling.

## Introduction

Volatile anesthetics are widely used for anesthesia or sedation during surgeries or diagnostic procedures across all age groups, including pediatric patients, due to their rapid onset and quick recovery. Sevoflurane (SEVO) is preferred for its minimal airway irritation and improved control over anesthesia depth, while isoflurane (ISO) is often chosen for its cost-effectiveness in prolonged procedures. However, exposure to anesthesia at a very young age may lead to long-term neurobehavioral impairments^1–3^. Previous animal studies have shown that anesthesia in neonatal rodents induces acute neuroapoptosis and subsequent cognitive deficits later in life^4–6^. Similar research in non-human primates has also indicated acute brain cell toxicity^7–9^ and long-term behavioral changes such as increased anxiety and reduced social behavior^10–12^.

While much is known about the neurotoxic effects of anesthetics on developing brains, there is no consensus on the mechanisms underlying anesthesia-induced developmental neurotoxicity. A significant concern is that changes in cerebral blood flow induced by anesthesia could reduce the supply of oxygen and nutrition to the brain^13,14^, potentially contributing to developmental neurotoxicity^15–17^. Under normal conditions, changes in vessel diameter play a crucial role in regulating blood flow to maintain optimal oxygen and nutrient delivery to the brain^18^. The adult brain can autonomously adjust to maintain adequate cerebral blood flow despite fluctuations in systemic blood pressure during anesthesia exposure^13,19,20^. Vasodilation, or the increase in vessel diameter, has been observed with most inhaled anesthetics, including SEVO and ISO, in both adult patients^21^ and animal models^22–24^. However, it remains unclear whether this vasodilatory response to anesthetics is fully developed in immature brains.

In this study, we investigated the effects of volatile anesthetics on cerebral vessel diameter in the cortex of mice at different developmental stages: postnatal day 13–14 (P13–14) for infants, P16–19 for juveniles, and P60–90 for adults. Using in vivo two-photon imaging, we observed that SEVO and ISO induce dilation of cortical arterioles and arteriole-proximate capillaries in an age-dependent manner, with the effects being most prominent in adults but reduced or absent in juveniles and infants. Through a combination of cell-type-specific Ca^2+^ imaging, optogenetic manipulation, and cell ablation techniques, our results indicate that anesthesia-induced vasodilation is mediated by neural/glial antigen 2 (NG2)-expressing vascular mural cells and Kir6.1, a subunit of ATP-sensitive potassium channels (K_ATP_) known for its vasodilatory function^25,26^. The lower density of vascular mural cells, particularly pericytes, and reduced expression of Kir6.1 during early postnatal development likely contributes to the observed lack of vasodilation in immature brains.

## Results

### SEVO induces dilation of cerebral arterioles but not venules in adult mice

**Fig. 1:**
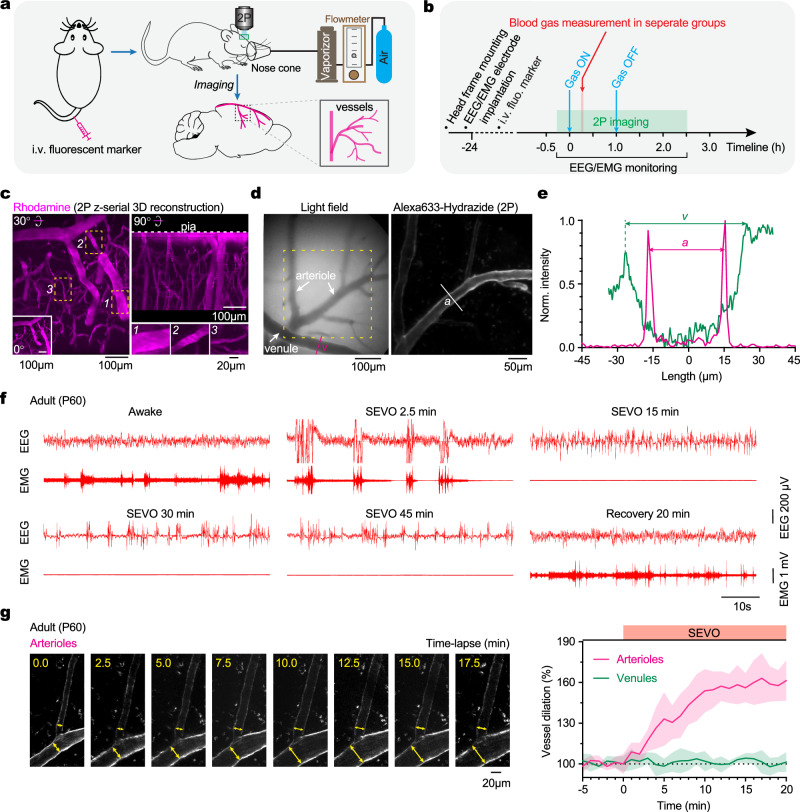
Exposure of adult mice to SEVO induces dilation of cerebral arterioles. **a** Schematic of in vivo two-photon imaging of cerebral vessels in the cortex of mice exposed to volatile anesthetics. **b** Experimental timeline for animal preparation, in vivo two-photon imaging, EEG/EMG recording, anesthesia exposure, and blood gas measurements. **c** Two-photon images of cortical vessels following rhodamine intravenous (i.v.) injection. Insets show boxed regions in higher magnification. **d** Left, light-field image showing a venule (*v*) and a branching arteriole. Right, two-photon image of an arteriole (*a*) stained by Alexa Fluor 633 hydrazide. **e** Light intensity profile across the line shown in (**d**) for measuring the diameter of cerebral arterioles and venules. Diameters are determined as the distance (µm) between light intensity peaks. **f** Representative EEG and EMG traces of an adult mouse before (awake), during, and after SEVO exposure. **g** Left, time-lapse images showing the dilation of cerebral arterioles (indicated by arrows) upon exposure of an adult mouse to SEVO 2.5%. Right, quantification of SEVO-induced vasodilation in arterioles (*n* = 73 from three mice) and venules (*n* = 56 from three mice) over time. Shading indicates 95% CI.

We used two-photon microscopy to observe changes in blood vessel diameter in the cerebral cortex of live mice before, during, and after exposure to volatile anesthetics (Fig. 1a, b). Following the implantation of a cranial window and electroencephalogram (EEG) and electromyogram (EMG) electrodes, awake mice received tail vein injections of either rhodamine to label the entire vasculature (Fig. 1c) or Alexa Fluor 633 hydrazide (Alexa633) (Fig. 1d), a dye that specifically binds to elastin in arteriole walls^27^. This method allowed us to visualize cerebral vessels ranging from 2 to 45 μm in diameter at depths up to 400 μm below the pial surface. Arteriole diameter was determined by measuring the distance between fluorescence intensity peaks across the vessel segment, while venule diameter was measured using bright-field images (Fig. 1e). Characteristic changes in EEG and EMG signals confirmed the induction, onset, and recovery from anesthesia (Fig. 1f). Our findings show that exposure of adult mice to SEVO resulted in a progressive increase in cerebral arteriole diameter, becoming noticeable within 3 min of exposure, peaking around 15 min, and plateauing thereafter (Fig. 1g). In contrast, there were no significant changes in venule diameter following SEVO exposure. These results indicate that SEVO induces dilation specifically in cerebral arterioles of adult mice.

### SEVO-induced arteriole dilation is absent in infant mice

**Fig. 2:**
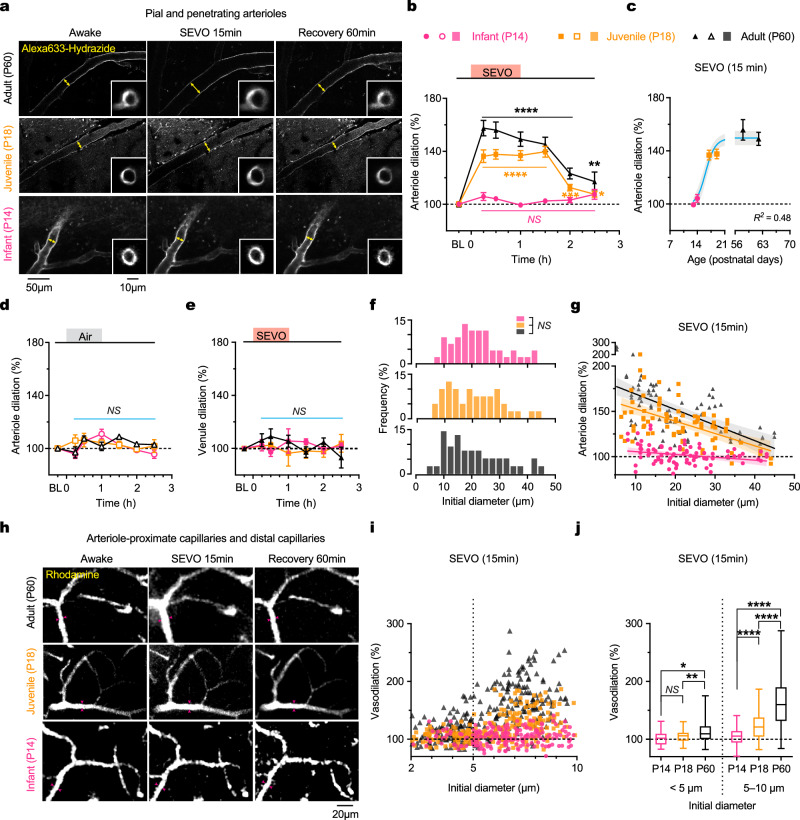
SEVO-induced vasodilation is robust in adult mice but reduced or absent in juveniles and infants. **a** Two-photon images of Alexa633 hydrazide-labeled cerebral pial arterioles and penetrating arterioles (inset) in P60, P18, and P14 mice before, 15 min after exposure to SEVO 2.5%, and 1 h after exposure. Arrows indicate changes in arteriole diameter. **b** Changes in arteriole diameter in mice of various ages before and after SEVO exposure. *n* = 76, 80, and 88 arterioles from five mice per group. BL, baseline, 100%; SEVO 15 min: 157.4 ± 5.9 (P60), 136.3 ± 4.8 (P18), and 105.9 ± 1.5 (P14). **c** Degree of SEVO-induced arteriole dilation positively correlates with the age of the animals (Sigmoidal model, *R*^*2*^ = 0.48, *P* < 0.0001). Each data point was calculated from 41 to 55 arterioles from two to three mice per age group. Shading, 95% CI. **d** Changes in arteriole diameter in mice of various ages before and after air exposure. *n* = 76, 80, and 88 arterioles from five mice per group. **e** Changes in venule diameter in mice of various ages before and after SEVO exposure. *n* = 68, 56, and 61 venules from five mice per group. **f** Distribution of initial diameters of arterioles sampled from mice of various ages (*n* = 76, 80, and 88 arterioles from five mice per group; *P* = 0.9642, 0.8154, 0.6077). **g** Relationship between the initial diameter of arterioles and the extent of dilation upon 15-min SEVO exposure. Data points represent 76, 80, and 88 individual arterioles from five mice and were fitted using a linear regression model (*R*^*2*^ = 0.36, 0.31 and 0.07; *P* < 0.0001, < 0.0001 and = 0.01; slope = −1.73, −1.37 and −0.34% µm^−^^1^ for adult, P18 and P14, respectively). Shading, 95% CI. **h** Two-photon images of rhodamine-filled capillaries in P60, P18, and P14 mice before, 15 min after exposure to SEVO 2.5%, and 1 h after exposure. Arrows indicate changes in vascular diameter. **i** Relationship between the initial vessel diameter (2–10 µm) and the extent of SEVO-induced vasodilation at 15 min (*n* = 199, 246, 298 individual vessels from 4, 5, 5 mice). **j** SEVO-induced dilation of capillaries at 15 min in P14, P18 and P60 mice (*P* = 0.3010, 0.0011, 0.0287, < 0.0001, < 0.0001, < 0.0001). Summary data are mean ± SEM. *P < 0.05, **P < 0.01, ***P < 0.001, ****P < 0.0001; *NS*, not significant; by two-tailed paired Student’s *t*-tests (**b**, **d**, **e**) or Kolmogorov-Smirnov tests (**f**, **j**).

To evaluate age-related variations in SEVO-induced cerebral arteriole dilation, we exposed infant (P14) and juvenile (P18) mice, alongside adults, to SEVO and monitored changes in vessel diameter over time. In adult cortex, SEVO triggered a substantial increase in arteriole diameter from the awake baseline (100%), peaking at 157.4 ± 5.9% (*P* < 0.0001) after 15 min and maintaining this dilation throughout the 60-min exposure (Fig. 2a–c). In P18 mice, arteriole diameter moderately increased to 136.3 ± 4.8% (*P* < 0.0001) at 15 min and remained stable thereafter (Fig. 2a–c). The degree of SEVO-induced arteriole dilation was significantly lower in P18 mice compared to adults at both 15 min (*P* < 0.0001) and 60 min (*P* < 0.0001) post-exposure initiation. After SEVO exposure ceased, arteriole diameter gradually returned to baseline in both adults (123.1 ± 4.1% at 60 min; 117.0 ± 7.2% at 90 min) and P18 mice (112.6 ± 2.4% at 60 min; 107.6 ± 2.2% at 90 min). Importantly, no significant changes in cerebral arteriole diameter were detected throughout SEVO exposure in infant mice (*P* > 0.05) (Fig. 2a–c). Air exposure for 60 min had no effect on arteriole diameter across all age groups (Fig. 2d). Similarly, venule diameter remained unchanged in younger mice before, during, and after SEVO exposure (Fig. 2e). These findings indicate an age-dependent pattern where SEVO-induced cerebral arteriole dilation is pronounced in adults but reduced or absent in juveniles and infants.

Further analysis of arterial trees in the mouse cortex revealed a consistent frequency distribution of arteriole sizes among the three age groups (Fig. 2f). SEVO-induced vasodilation was more pronounced in smaller arterioles, with the extent of dilation decreasing linearly with increasing initial diameter (Adult, *R*^2^ = 0.36, *P* < 0.0001; P18, *R*^2^ = 0.31, *P* < 0.0001; P14, *R*^2^ = 0.07, *P* = 0.01) (Fig. 2g). This suggests that SEVO preferentially affects smaller arterioles in the cortex.

Since Alexa633-labeled arterioles typically have a minimum diameter of ~10 µm^27^, we further examined the effects of SEVO on vessels in the arteriole-capillary transitional zone^28,29^ (arteriole-proximate capillaries) and distal capillaries using rhodamine injection (Fig. 2h; Supplementary Fig. 1). Similar to arterioles with initial diameters greater than 10 µm (Fig. 2g), we observed age-dependent vasodilation in microvessels with diameters between 5 and 10 µm during SEVO exposure (Fig. 2i, j). In contrast, distal capillaries (<5 µm) exhibited a heterogeneous response to anesthesia, with only a subset showing dilation upon SEVO exposure in adult mice (Fig. 2i, j). These findings are consistent with previous observations that pericytes can induce more substantial and rapid diameter changes in proximal capillaries compared to distal capillaries^30,31^.

### ISO induces age-dependent vasodilation similar to SEVO

**Fig. 3:**
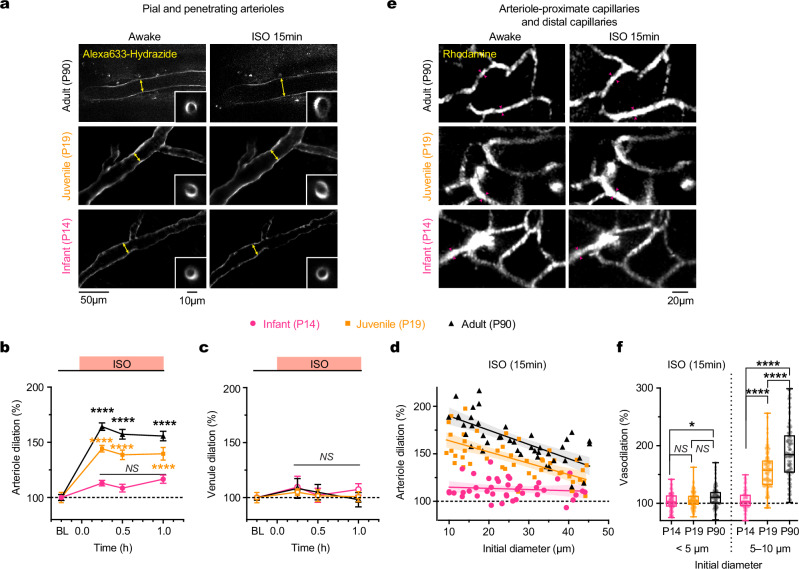
Age-dependent dilation of cortical arterioles in mice exposed to ISO. **a** Representative two-photon images of Alexa633 hydrazide-labeled cerebral pial arterioles and penetrating arterioles (inset) in adult, P19, and P14 mice before and 15 min after exposure to ISO 1.5%. Arrows indicate changes in arteriole diameter. **b** Changes in arteriole diameter over time after exposure to ISO in adult, P19, and P14 mice (*n* = 43, 47 and 41 arterioles from three mice per group; *P* < 0.0001 for adult and P19 mice). BL, baseline under awake conditions. ISO 15 min: adult, 164.3 ± 3.4; P19, 144.2 ± 3.0; P14, 113.0 ± 1.6. **c** Changes in venule diameter over time after exposure to ISO in adult, P19, and P14 mice (*n* = 46, 44 and 51 venules from three mice per group). ISO 15 min: adult, 108.3 ± 9.0; P19, 105.1 ± 3.1; P14, 109.5 ± 9.9. **d** Relationship between the initial diameter of arterioles and the extent of dilation at 15 min after ISO exposure in adult, P19, and P14 mice (*n* = 43, 47 and 41 arterioles from three mice per group). Data were fitted with a linear regression model (*R*^*2*^ = 0.52, 0.46, and 0.013; *P* < 0.0001, < 0.0001, and = 0.48; slope = −1.50, −1.19 and −0.13% µm^−^^1^ for adult, P19 and P14, respectively). **e** Two-photon images of rhodamine-filled capillaries in adult, P19, and P14 mice before and 15 min after exposure to ISO 1.5%. Arrows indicate changes in arteriole diameter. **f** ISO-induced dilation of capillaries (< 5 µm, *n* = 95, 97, 91 vessels from three mice per group, *P* = 0.4695, 0.0691, 0.0123; 5–10 µm, *n* = 80, 119, 116 vessels from three mice per group, *P* < 0.0001 for all) at 15 min in P14, P19 and adult mice. Data are mean ± SEM. *P < 0.05, ****P < 0.0001; *NS*, not significant; by two-tailed paired Student’s *t*-tests (**b**, **c**) or Kolmogorov-Smirnov tests (**f**).

In addition to SEVO, we investigated the effects of ISO on cerebral vessel diameters in vivo following the experimental protocol outlined in Fig. 1b. Infant (P14), juvenile (P19), and adult mice were exposed to ISO for 1 h. Similar to SEVO, ISO exposure induced significant dilation of cortical arterioles in adult and juvenile mice, peaking at 15 min (Adult, 164.3 ± 3.4%, *P* < 0.0001; P19, 144.2 ± 3.0%, *P* < 0.0001) and maintaining this level throughout the exposure (Fig. 3a, b). In contrast, cortical arterioles in infant mice showed only a slight trend toward dilation (113.0 ± 1.6%, *P* = 0.27, at 15 min), which did not reach statistical significance compared to awake conditions. Similar to SEVO, ISO had no effect on the diameter of cortical venules in infant, juvenile, and adult mice (Fig. 3c).

In adult mice, ISO’s vasodilatory effects were more pronounced in smaller arterioles and decreased linearly with increasing initial diameter (*R*^2^ = 0.52, *P* < 0.0001) (Fig. 3d). Additionally, we observed an age-dependent vasodilatory effect of ISO on arteriole-proximate capillaries (5–10 µm), but not on distal capillaries (< 5 µm) (Fig. 3e, f). Collectively, these results indicate that both ISO and SEVO induce age-dependent vasodilation in cortical arterioles and their proximate capillaries.

### Measurements of physiological parameters

To understand the age-dependent vasodilatory response to volatile anesthetics, we first assessed respiratory rates and arterial blood gas parameters in different age groups during SEVO exposure. After 15 min of exposure, when arteriole dilation had plateaued in adult and juvenile mice, we observed mild acidosis across all age groups (Supplementary Table 1). Arterial pCO_2_ levels were within the normal range in adult mice (40.9 ± 1.6 mmHg) but elevated in infants (64.7 ± 1.9 mmHg). Given that hypercapnia is a significant physiological stimulus for vasodilation, the reduced SEVO-induced vasodilation in younger mice is unlikely to be attributed to alterations in blood gases.

### NG2^+^ mural cells are less abundant in the developing cortex

At the cellular level, changes in cerebral vessel diameter are regulated by vascular mural cells, including vascular smooth muscle cells (vSMCs) and pericytes. vSMCs, identified by their ring-like morphology and expression of α-SMA, cover pial arterioles and the proximal segments of penetrating arterioles. Pericytes in the arteriole-capillary transitional zone have an ensheathing morphology, whereas those around distal capillaries possess elongated processes that span up to several vessel branches^28,32^.

**Fig. 4:**
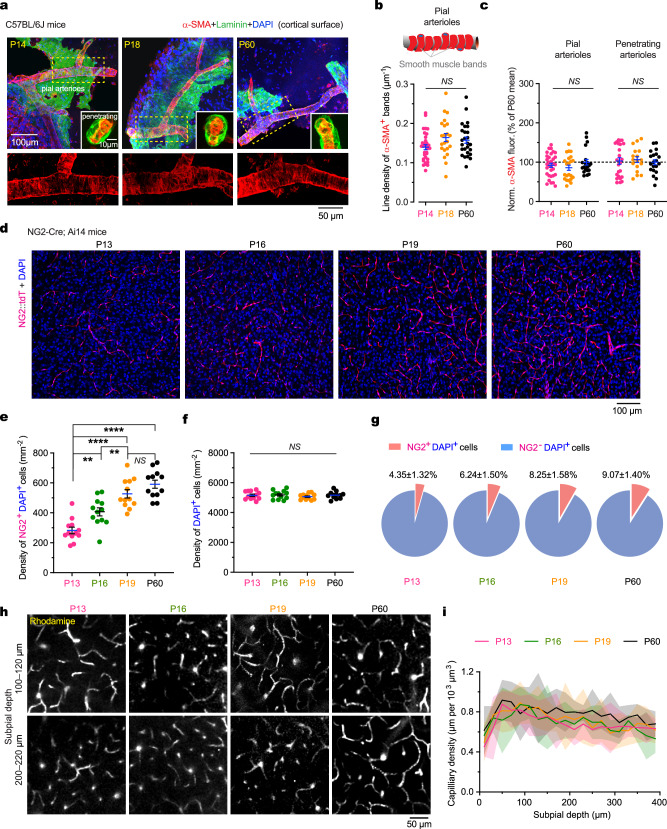
NG2^+^ pericytes are less abundant in the cortex of infant mice than in adults. **a** Immunofluorescence images of transverse sections of the cortex showing pial arterioles in mice of various ages, triple-stained for α-SMA (red), laminin (green), and DAPI (blue). Insets show penetrating arterioles at higher magnification. Boxed regions are enlarged below, displaying α-SMA staining of pial arterioles. **b** Density of α-SMA^+^ bands in pial arterioles (*n* = 32, 22, and 26 arterioles from three mice per group; *P* = 0.2537, 0.9044, 0.3663). P14, P18, and P60 (×10^−^^3^): 139.7 ± 6.5, 164.2 ± 10.3, and 157.3 ± 8.1. **c** Normalized α-SMA fluorescence intensity in pial and penetrating arterioles. Pial arterioles: P14, 92.2 ± 5.2, *n* = 32; P18, 86.0 ± 6.7, *n* = 22; P60, 100.0 ± 7.2, *n* = 26. *P* = 0.3891, 0.0700, 0.7085. Penetrating arterioles: P14, 103.1 ± 8.3, *n* = 24; P18, 106.7 ± 7.4, *n* = 17; P60, 100.0 ± 6.5, *n* = 20. *P* = 0.6533, 0.8308, 0.5925. **d** Confocal images of the mouse cortex showing tdTomato (tdT)-expressing NG2^+^ cells and DAPI stained nuclei. **e**, **f** Density of NG2^+^DAPI^+^ cells (**e**) and DAPI^+^ cells (**f**) in the cortex at different ages. **g** Percentage of NG2^+^ cells in the mouse cortex at different ages (*n* = 12 slices from four mice per group). *P* = 0.0018, 0.0068, 0.0780, and < 0.0001 in (**e**), *P* = 0.8874, 0.3777, 0.3474, 0.9774 in (**f**). **h** Two-photon Z-stack images of rhodamine-filled capillaries collected from depths of 100–120 µm or 200–220 µm in the cortex of mice at different ages. **i** Quantification of capillary density (total length per parenchymal volume) across varying depths of the mouse cortex at different ages (*n* = 3 mice per group; *F*_(57, 160)_ = 1.15, *P* = 0.2480). Shading, 95% CI. Data are mean ± SEM. **P < 0.01, ****P < 0.0001; *NS*, not significant; by two-tailed Kolmogorov-Smirnov tests (**b**, **c**), two-tailed Mann-Whitney *U* tests (**e**, **f**), or two-way ANOVA tests (**i**).

We performed immunohistochemistry (IHC) analysis of α-SMA and NG2 in the cortex of infant, juvenile, and adult mice (Fig. 4). Our comparison of these age groups revealed no significant differences in the density of vSMCs (estimated from α-SMA^+^ band density) covering pial arterioles (Fig. 4a, b) or in α-SMA expression in pial arterioles and the proximal segments of penetrating arterioles (Fig. 4a, c). However, the density of NG2^+^ cells with pericyte morphology was approximately two-fold lower in the cortex of infant mice compared to juveniles and adults (P13, 282.1 ± 22.1; P16, 406.7 ± 27.0; P19, 527.0 ± 28.1; P60, 591.2 ± 27.1 cells mm^−^^2^) (Fig. 4d, e; Supplementary Figs. 2 and 3). Additionally, the percentage of NG2^+^ cells relative to all cortical cells increased from 4.4% in P13 mice to 9.1% in adult mice (Fig. 4f, g). Analysis of capillary densities at various subpial depths showed no significant differences among infant, juvenile, and adult mice (Fig. 4h, i). Together, these results indicate that the developing cortex has a lower density of NG2^+^ mural cells with pericyte morphology, which may contribute to the reduced vasodilatory response observed in younger mice exposed to volatile anesthetics.

### SEVO decreases cytosolic Ca^2+^ in NG2^+^ mural cells

Mural cells, including vSMCs and pericytes, play an important role in regulating cerebral microvessel diameter in response to neuronal and astrocytic signals^30,31,33^. Changes in their intracellular Ca^2+^ concentrations have been associated with vasoconstriction and vasodilation^34^. To investigate the role of NG2^+^ mural cells in anesthesia-induced arteriole dilation, we crossed NG2-Cre mice with Ai14 reporter mice to express the red fluorescent protein tdTomato in NG2^+^ cells. This confirmed substantial colocalization of NG2^+^ cells with the cortical vasculature, as visualized through intravenous (i.v.) injection of FITC (Supplementary Fig. 4). These results indicate that the majority of cortical NG2^+^ cells are vascular mural cells with pericyte morphology.

**Fig. 5:**
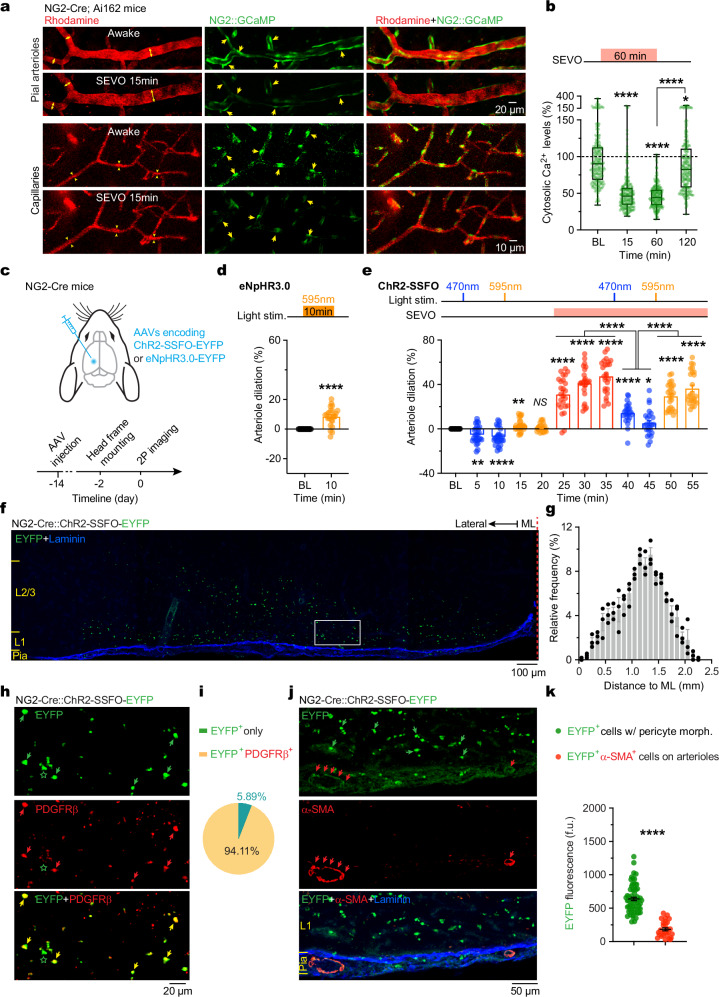
Ca^2+^ imaging and optogenetic stimulation reveal the role of NG2^+^ cells in regulating arteriole diameter in adult mice. **a** Two-photon images showing GCaMP-expressing NG2^+^ cells and rhodamine-filled pial arterioles (upper) and capillaries (lower; at 200 µm depth) in the cortex of adult mice before and 15 min after SEVO exposure. Arrows indicate NG2^+^ cells with pericyte morphology. **b** Normalized Ca^2+^ concentrations in NG2^+^ pericytes before, 15 and 60 min after SEVO exposure, and 60 min after emergence from SEVO (*n* = 225 cells from four adult mice; *P* = 0.0206, < 0.0001). BL, baseline under awake conditions. **c** Experimental design for optogenetic inhibition (eNpHR3.0) or activation (ChR2-SSFO) of cortical NG2^+^ cells. **d** Changes in arteriole diameter (8.4 ± 1.3%; *n* = 23 from three mice) following light stimulation (595 nm, 10 min) in adult mice expressing eNpHR3.0 in NG2^+^ cells (*P* < 0.0001). **e** Changes in arteriole diameter (*n* = 25 from three mice) in adult mice expressing ChR2-SSFO in NG2^+^ cells in response to blue (470 nm) or yellow (595 nm) light stimulation and SEVO exposure, as indicated. In awake mice, blue light activation of NG2^+^ cells induced vasoconstriction at 5 and 10 min, which was reversed by subsequent yellow light deactivation at 15 min and 20 min. SEVO exposure caused robust vasodilation that persisted until 35 min. Activation of NG2^+^ cells during SEVO led to arteriole contraction, which was reversed by deactivation (*P* = 0.0022, 0.0088, 0.1386, 0.0377, and < 0.0001 for others). **f** Confocal image of the cortex showing NG2^+^ cells expressing EYFP (green) and the pial surface immunostained for laminin (blue). ML, midline. Boxed region is shown in (**h**). **g** Distribution of EYFP-expressing NG2^+^ cells in the cortex (*n* = 3 mice). ML, midline. **h** Confocal image of the cortex showing NG2^+^ cells expressing EYFP (green), immunostained for PDGFRβ (red). Arrows and stars indicate NG2^+^ cells positive or negative for PDGFRβ. **i** Percentage of NG2^+^ cells with or without PDGFRβ expression (9 slices from three mice). **j** Similar to (**h**), but immunostained for α-SMA (red) and laminin (blue). Arrows indicate cerebral arterioles expressing high levels of α-SMA. **k** Quantification of EYFP levels in smooth muscle cells (indicated by arrows in **j**) and cells with pericyte morphology (*P* < 0.0001). Data are mean ± SEM. *P < 0.05, **P < 0.01, ****P < 0.0001; *NS*, not significant; by two-tailed paired *t-*tests (**b**, **d**, **e**) or Kolmogorov-Smirnov tests (**g**, **k**).

Next, NG2-Cre mice were bred with Ai162 mice to express the genetically encoded Ca^2+^ indicator GCaMP6s in NG2^+^ cells. We performed in vivo Ca^2+^ imaging in the cortex of adult mice before and after SEVO exposure. In awake mice, cytosolic Ca^2+^ was readily observed in vascular mural cells, including those with pericyte morphology (Fig. 5a). The level of cytosolic Ca^2+^ significantly decreased 15 min after SEVO exposure (50.3 ± 1.7%, *P* < 0.0001) compared to the pre-exposure baseline (100%). This reduction persisted throughout SEVO exposure (45.9 ± 1.0%, *P* < 0.0001, at 60 min) and returned to baseline 60 min after SEVO exposure ended (88.6 ± 2.5%, *P* < 0.0001, 120 min *vs*. 60 min) (Fig. 5b). This pattern of Ca^2+^ reduction correlated with SEVO-induced arteriole dilation and subsequent recovery (Fig. 2b), suggesting that Ca^2+^ signaling in NG2^+^ mural cells is a key factor in arteriole relaxation during anesthetic exposure in adult mice.

### Optogenetic stimulation of NG2^+^ cells modulates vascular tone

We used optogenetic techniques to manipulate NG2^+^ cells in the cortex of adult mice and observed changes in vessel diameter using two-photon microscopy (Fig. 5c). To inhibit (hyperpolarize) NG2^+^ cells, we stereotaxically injected adeno-associated viruses (AAVs) encoding Cre-dependent halorhodopsins (eNpHR3.0) into the cortex of NG2-Cre mice, resulting in the expression of a light-driven chloride pump specifically in NG2^+^ cells. Two weeks after viral injection, continuous yellow light stimulation (595 nm, 10 min) induced a significant increase in the diameter of cortical arterioles in awake mice (Fig. 5d). These results indicate that optogenetic inhibition of NG2^+^ cells can induce dilation of cortical arterioles in non-anesthetized mice.

In a separate cohort, we used stabilized step-function channelrhodopsins (ChR2-SSFO) to activate (depolarize) cortical NG2^+^ cells in a reversible manner (Fig. 5c). ChR2-SSFO remains open for over 30 min following brief blue light (470 nm) stimulation and can be closed immediately with a yellow light pulse^35^. In awake mice, blue light stimulation caused a significant decrease in the diameter of cortical arterioles, which was promptly reversed by yellow light (Fig. 5e). Subsequent SEVO exposure led to substantial dilation of these arterioles, which was then effectively reversed by another blue light pulse, reactivating NG2^+^ cells, followed by dilation upon yellow light stimulation (Fig. 5e). These results demonstrate that optogenetic activation of NG2^+^ cells can induce constriction of cortical arterioles in adult mice, both during and outside SEVO exposure.

To verify the identity of NG2^+^ cells expressing ChR2-SSFO-EYFP (Fig. 5f, g), we performed immunostaining for platelet-derived growth factor β (PDGFRβ), a marker for both pericytes and vSMCs^36,37^. Our analysis revealed that more than 94% of NG2^+^ cells (EYFP^+^) in the cortex were positive for PDGFRβ (Fig. 5h–k), confirming that these cells are predominantly pericyte-like mural cells. Crossbreeding NG2-Cre mice with Ai14 reporter mice further confirmed that the viral infection predominantly transduced ChR2-SSFO-EYFP into mural cells with pericyte morphology (Supplementary Fig. 5).

### Ablation of NG2^+^ cells eliminates SEVO-induced arteriole dilation

**Fig. 6:**
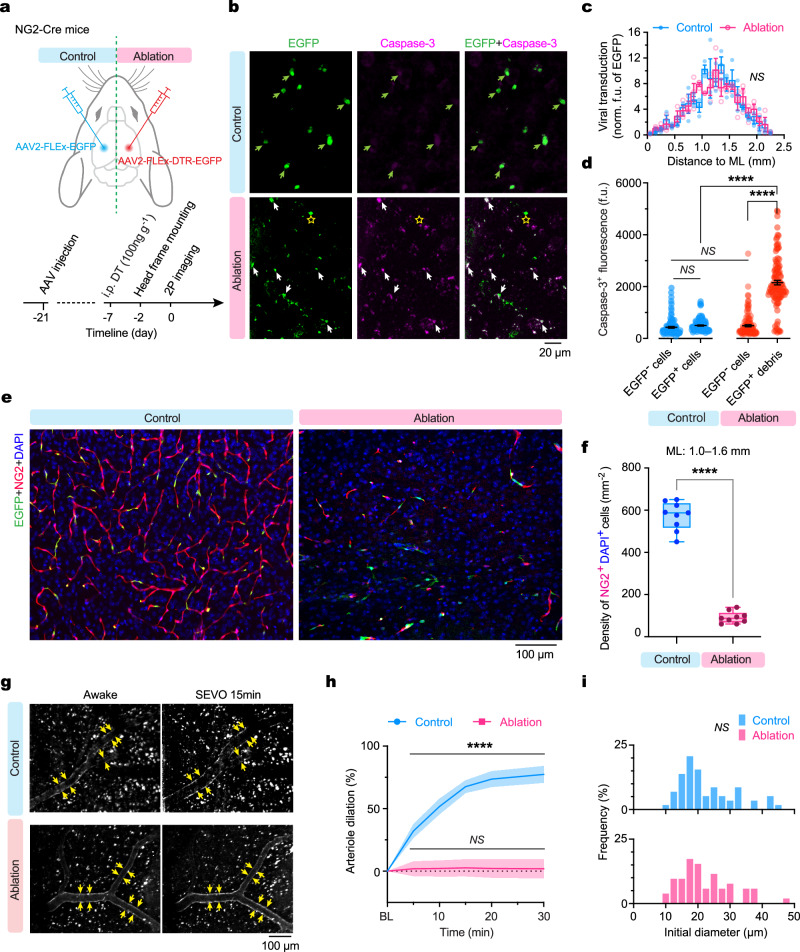
Genetic ablation of NG2^+^ cells abolishes SEVO-induced arteriole dilation in the cortex of adult mice. **a** Experimental design for spatially confined ablation of cortical NG2^+^ cells. NG2-Cre mice were injected with AAV2-FLEx-DTR-EGFP and AAV2-FLEx-EGFP (control) into the right and left cortex, respectively, on day −21. An i.p. injection of diphtheria toxin (DT) on day −7 ablated DTR-expressing NG2^+^ cells, and two-photon imaging on day 0 assessed changes in arteriole diameter upon SEVO 2.5%. **b** Images of the cortex stained for cleaved caspase-3. In the control cortex, EGFP^+^ cells (green arrows) express low levels of cleaved caspase-3; in the ablation cortex, most DTR-EGFP^+^ cells show high caspase-3 expression (white arrows), with a few exceptions (yellow star). **c** Viral transduction range measured by distribution of EGFP^+^ fluorescence intensity (f.u., fluorescence unit) in the control (EGFP) and ablation (DTR-EGFP) cortex (*n* = 3 mice per group; *F*_(22, 88)_ = 1.278, *P* = 0.2096). ML, midline. **d** Expression of cleaved caspase-3 measured by fluorescent intensity in either side of the cortex (9 slices from three mice; from left to right, *n* = 107, 102, 123, and 118 cells; *P* = 0.0856, 0.2568, and < 0.0001 for others). **e** Immunofluorescence images of the cortex stained for virally transduced EGFP (green), antibody-labeled NG2 (red), and DAPI (blue) from the control and ablation cortex. **f** Density of NG2^+^DAPI^+^ cells in the control and ablation cortex (*n* = 12 slices from three mice per group; *P* < 0.0001). **g** Two-photon images of cortical arterioles before and 15 min after SEVO exposure. Arrows indicate SEVO-induced dilation of arterioles in the control (EGFP) but not the ablation (DTR-EGFP) cortex. **h** Time course of SEVO-induced arteriole dilation in the control (*n* = 64 segments from four mice) and ablation (*n* = 58 segments from four mice) cortex of adult mice exposed to SEVO at time 0. BL, baseline under awake conditions. **i** Distribution of initial diameters of the sampled arterioles. There is no difference between the control and ablation cortex (*P* = 0.9924). ****P < 0.0001; *NS*, not significant; by two-way ANOVA (**c**), two-tailed unpaired *t-*tests (**d**), two-tailed Mann-Whitney *U* test (**f**), two-tailed paired *t*-tests (**h**), or Kolmogorov-Smirnov test (**i**).

To validate the role of NG2^+^ mural cells in SEVO-induced vasodilation in the adult cortex, we employed diphtheria toxin (DT)-mediated cell ablation^38^. We stereotaxically injected AAV expressing Cre-dependent diphtheria toxin receptor (DTR)-EGFP into the cortex of NG2-Cre mice on one side. Simultaneously, AAV encoding EGFP only was injected into the contralateral cortex of the same animals as a control. Two weeks post-viral injection, we administered a single dose of DT to induce cell ablation (Fig. 6a). Comparison of the control cortex, where NG2^+^ cells expressed EGFP only, with the ablation cortex, where NG2^+^ cells expressed DTR-EGFP, showed a significant increase in apoptosis (Fig. 6b–d) and a marked reduction in NG2^+^ cells (Fig. 6e, f) in the latter, confirming effective depletion of cortical NG2^+^ cells.

Following SEVO exposure, we observed a striking contrast in vasodilatory responses between the control and ablation cortices (Fig. 6g, h). In the control cortex with intact NG2^+^ cells, SEVO exposure induced a substantial increase in cerebral arteriole diameter, consistent with findings in wild-type mice (Fig. 2b). In contrast, SEVO-induced arteriole dilation was completely abolished in the cortex depleted of NG2^+^ cells. The distribution of initial diameters of cortical arterioles in awake mice before SEVO exposure remained unchanged following NG2^+^ cell ablation (Fig. 6i). These results underscore the critical role of NG2^+^ mural cells in mediating SEVO-induced cerebral vasodilation and offer insights into the reduced vasodilatory response to anesthetics observed in immature brains, where vascular mural cells, particularly pericytes, are less abundant.

### Kir6.1 mediates age-dependent vasodilatory responses to SEVO

To investigate the molecular mechanisms underlying age-dependent vasodilatory responses to volatile anesthetics, we focused on Kir6.1, a subunit of K_ATP_ encoded by the *Kcnj8* gene. Previous studies have shown that pharmacological blockade of K_ATP_ can eliminate cerebral vasodilation induced by volatile anesthetics^25^. Recent single-cell transcriptomic research has also highlighted that Kir6.1 is highly expressed in pericytes compared to other cell types in the mouse brain^26^. We hypothesized that Kir6.1 signaling contributes to the age-dependent vasodilatory responses to volatile anesthetics mediated by NG2^+^ mural cells.

**Fig. 7:**
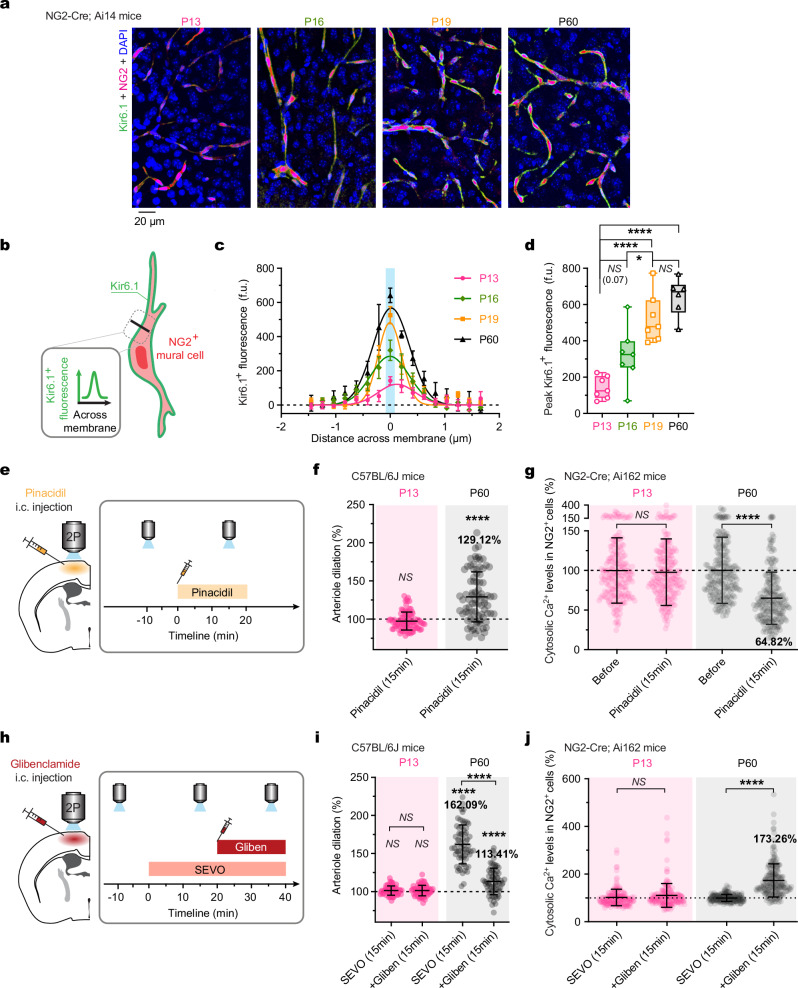
NG2^+^ mural cells contribute to age-dependent cerebral vasodilation via Kir6.1-dependent mechanisms. **a** Confocal images of cortical NG2^+^ cells expressing tdTomato, immunostained for DAPI (blue) and Kir6.1 (green) at different ages. Note the increasing Kir6.1 fluorescence from P13 to P60. **b** Schematic depicting the quantification of Kir6.1 expression levels on the NG2^+^ cell membrane. Peak fluorescence intensity of Kir6.1 across the cell membrane is measured to indicate expression levels. **c** Quantification of Kir6.1 fluorescence intensity across the cell membrane (*n* = 7, 7, 7, 6 mice per group). **d** Peak fluorescence intensity of Kir6.1 measured from (**c**). *F*_(3, 23)_ = 22.23, *P* = 0.0677, 0.0243, 0.5876, and < 0.0001 for others. **e**, **h** Schematic illustrating the experimental design for intracortical (i.c.) injection of pinacidil (a K_ATP_ channel opener) or glibenclamide (a K_ATP_ channel inhibitor). **f**, **g** Injection of pinacidil during wakefulness induced significant cerebral vasodilation (*n* = 88 and 83 arterioles from four mice per group; *P* = 0.0510, < 0.0001) (**f**) and a decrease in Ca^2+^ activity of NG2^+^ cells (*n* = 211 and 218 cells from three mice per group; *P* = 0.3005, < 0.0001) (**g**) in adults (P60) but not in infant mice (P13). **i**, **j** Injection of glibenclamide during SEVO exposure attenuated arteriole dilation (*n* = 61 and 67 arterioles from three mice per group; *P* = 0.0539, 0.0987, 0.9269, and < 0.0001 for others) (**i**) and increased Ca^2+^ activity in NG2^+^ cells (*n* = 189 and 204 cells from three mice per group; *P* = 0.0610, < 0.0001) (**j**) in adults (P60) but not in infant mice (P13). Data are mean ± SEM (**c**) or ± SD (**f**, **g**, **i**, **j**). *P < 0.05, ****P < 0.0001; *NS*, not significant; by one-way ANOVA followed by Bonferroni’s comparisons (**d**) or two-tailed paired *t-*tests (**f**, **g**, **i**, **j**).

To test this hypothesis, we performed immunostaining for Kir6.1 in the cortex of NG2-Cre;Ai14 mice at various ages (Fig. 7a). Our results indicate an increasing expression of Kir6.1 in NG2^+^ mural cells from P13 to P19, with peak expression in adulthood (P60) (Fig. 7b–d). Due to the limitations of immunofluorescence for quantitative measurement of Kir6.1, we complemented this with pharmacological studies to examine its role in SEVO-induced arteriole dilation and alterations in cytosolic Ca^2+^ levels in NG2^+^ mural cells.

Under awake conditions, local activation of Kir6.1 by intracortical (i.c.) injection of pinacidil (Fig. 7e) induced significant arteriole dilation in adult mice (129.1 ± 3.6% in diameter) but not in P13 mice (97.5 ± 1.3%) (Fig. 7f). Similarly, Kir6.1 activation led to a reduction in cytosolic Ca^2+^ levels of NG2^+^ mural cells in adults (to 64.8 ± 2.2%) but not in P13 mice (100.0 ± 2.8%) (Fig. 7g). Conversely, local inhibition of Kir6.1 by i.c. injection of glibenclamide (Fig. 7h) significantly reduced SEVO-induced dilation (Fig. 7i) and increased cytosolic Ca^2+^ levels (Fig. 7j) in adults, with no significant effects in P13 mice. These findings support the involvement of Kir6.1 in SEVO-induced vasodilation mediated by NG2^+^ mural cells.

**Fig. 8:**
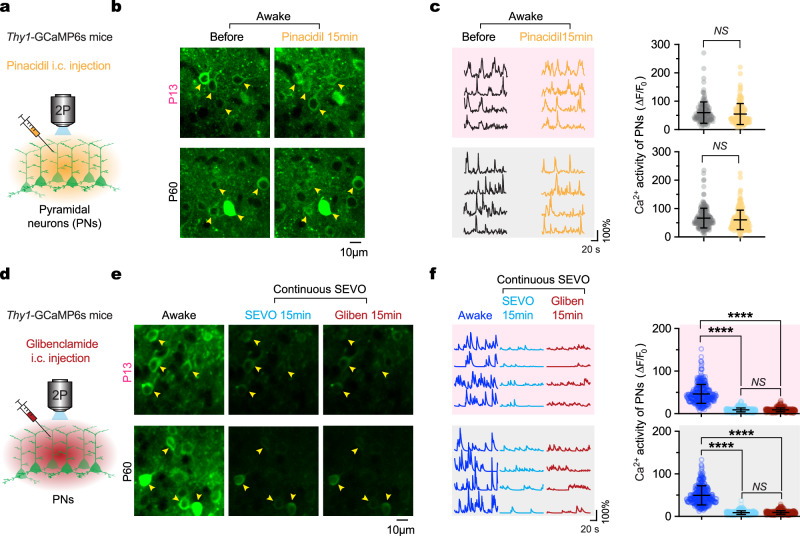
Pharmacological manipulation of Kir6.1 has no effect on Ca^2+^ activity of cortical pyramidal neurons during wakefulness or SEVO exposure. **a** Experimental design for in vivo Ca^2+^ imaging of cortical pyramidal neurons (PNs) before and after i.c. injection of pinacidil, a Kir6.1 opener. **b** Representative two-photon images of cortical pyramidal neurons expressing GCaMP in awake mice at P13 and P60, both before and 15 min after pinacidil injection. **c** Representative Ca^2+^ traces (left) and average spontaneous Ca^2+^ activity (right) of individual neurons before and after pinacidil administration (*n* = 141 and 204 cells from three to four mice per group; *P* = 0.1441, 0.0695). **d**–**f** Similar to (**a**–**c**), but for mice initially awake, subsequently exposed to SEVO, followed by i.c. injection of glibenclamide, a K_ATP_ inhibitor (*n* = 328 and 359 cells from five mice per group; *P* = 0.4610, 0.1101, and < 0.0001 for else). Data are mean ± SD. ****P < 0.0001; *NS*, not significant; by two-tailed paired *t-*tests (**c**, **f**).

In a separate experiment using *Thy1*-GCaMP6s transgenic mice, we examined the impact of Kir6.1 activation or inhibition on the Ca^2+^ activity of cortical pyramidal neurons. Neither activation nor inhibition of Kir6.1 produced significant changes in neuronal Ca^2+^ activity in adults or P13 mice (Fig. 8a–f), ruling out a potential role of neuronal activity^39^ in Kir6.1-mediated vasodilatory responses to SEVO.

## Discussion

**Fig. 9:**
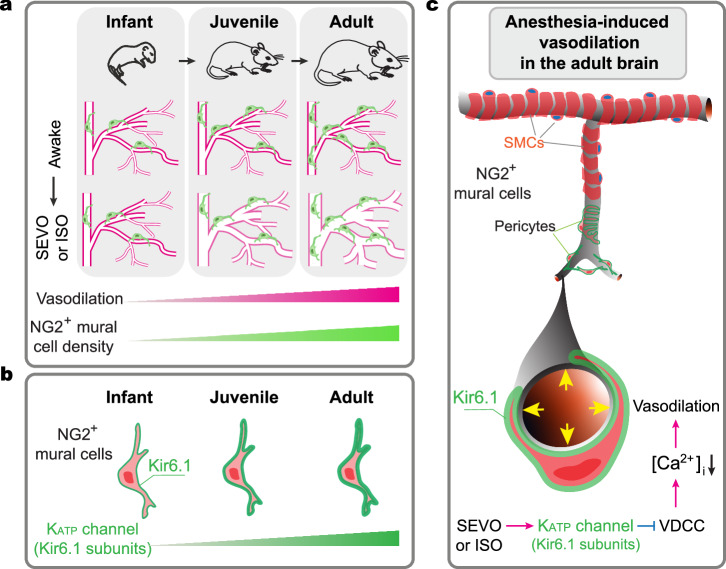
Schematic summary of age-dependent cerebral vasodilation during anesthesia. **a** Age-dependent vasodilatory responses to volatile anesthetics are correlated with the density of mural cells in the mouse cortex. **b** Expression of Kir6.1, a subunit of vasodilatory K_ATP_ channels, in NG2^+^ mural cells increases progressively during postnatal development. **c** Putative pericytes mediate anesthesia-induced vasodilation. Exposure to SEVO or ISO activates Kir6.1, which inhibits voltage-dependent Ca^2+^ channels (VDCCs), reducing intracellular Ca^2+^ concentration ([Ca^2+^]_i_) in NG2^+^ mural cells. This reduction in [Ca^2+^]_i_ leads to the dilation of penetrating arterioles and arteriole-proximate capillaries.

Exposure to volatile anesthetics induces cerebral vasodilation in adult animals^21,24^, but it is unclear whether this response occurs in the immature brain. Using in vivo time-lapse imaging of cerebral vasculature, we found that SEVO and ISO cause dilation of cortical arterioles in an age-dependent manner: most pronounced in adults, less so in juveniles, and minimal in infant mice. This vasodilation was particularly evident in smaller arterioles and at the arteriole-capillary transition, but not observed in venules. Through in vivo Ca^2+^ imaging, optogenetic stimulation, and cell ablation, we demonstrated that NG2^+^ mural cells mediate SEVO-induced arteriole dilation in the adult cortex via Kir6.1-dependent mechanisms. The lower density of pericyte-like mural cells and reduced Kir6.1 expression in the immature brain likely contributes to the inadequate cerebral vasodilation observed in infant mice (Fig. 9).

Anesthesia induces significant hemodynamic changes, and the adult brain can autonomously compensate for decreases in systemic blood pressure to maintain adequate cerebral blood flow during anesthesia. In adults, a decrease in perfusion pressure triggers vasodilation in cerebral arterioles to increase blood flow^19^. However, this autoregulatory response is not observed in very young animals^40,41^. Reduced cerebral autoregulation in healthy neonates suggests that this age group may be more vulnerable to decreases in cerebral perfusion and blood pressure during anesthesia^42,43^. Previous studies have shown variations in cerebrovascular reactivity depending on developmental stage; for example, cerebrovascular CO_2_ responsiveness is more pronounced in adults compared to fetal and newborn sheep^44^. Consistent with these findings, our study revealed an age-dependent vasodilatory response to inhaled anesthetics, being robust in adults but attenuated or absent in juveniles and infants. The absence of vasodilatory responses to anesthesia in the infant brain may result in inadequate cerebral blood flow. Prolonged insufficiency could lead to critical metabolic shortages of oxygen and nutrients, ultimately causing brain cell death.

Exposure of immature brains to anesthetics can result in neurotoxicity, leading to widespread neuronal apoptosis and lasting behavioral and cognitive impairments^1–3^. The period of rapid synaptogenesis is recognized as the time of greatest susceptibility to anesthetic-induced neurotoxic effects. In rodents, this vulnerable window generally occurs during the first 2 weeks of postnatal life^45^, with most long-term functional impairments observed in those exposed to anesthetics between P7–14^5,6,46,47^. Our study observed an absence of cerebral vasodilatory responses to SEVO and ISO in P13–14 mice, aligning with this critical period of heightened vulnerability. Future research is needed to investigate whether the lack of vasodilatory responses to anesthesia in the developing brain contributes to anesthesia-induced developmental neurotoxicity.

The age-dependent effects of anesthesia on cerebral vasodilation cannot be attributed to hypercapnia. In our study, we measured arterial blood gases after 15 min of SEVO exposure. The analyses revealed mild acidosis across all age groups of mice, consistent with prior reports of short or moderate periods of anesthesia exposure under spontaneous breathing in both infant^15,48^ and adult mice^49,50^. Notably, our assessments showed elevated CO_2_ levels in infants, a potent vasodilator, but not in adult mice. Despite this, infant mice did not exhibit a measurable vasodilatory response to SEVO or ISO, suggesting that changes in CO_2_ levels are unlikely to account for the age-related differences in vasodilatory responses to anesthesia. However, elevated blood CO_2_ may influence brain activity^51^. Future research should explore the potential role of mild hypercapnia in developmental neurotoxicity.

Our study highlights the pivotal role of NG2^+^ mural cells (putative pericytes) in mediating SEVO-induced cerebral vasodilation. This was demonstrated through a series of in vivo experiments using NG2-Cre mice for cell-type-specific Ca^2+^ imaging, optogenetic manipulation, and local ablation. First, IHC revealed a reduced number of NG2^+^ mural cells in the cortex of infant mice, which exhibited diminished vasodilatory responses. Second, exposure to SEVO in adult mice resulted in decreased cytosolic Ca^2+^ concentrations in NG2^+^ mural cells, affecting their contractile activity^34^. Third, optogenetic activation and inhibition of cortical NG2^+^ cells bidirectionally altered the diameter of cerebral arterioles, indicating a direct role of these cells in regulating vascular tone and the dilatory response to SEVO. Fourth, SEVO-induced vasodilation was entirely abolished in the cortex depleted of NG2^+^ cells, further supporting the essential role of NG2^+^ mural cells in this process.

Anesthesia-induced vasodilation is most prominent in smaller arterioles, such as distal penetrating arterioles, and at the arteriole-capillary transition zone. These vessels are surrounded by vSMCs and/or contractile ensheathing pericytes (α-SMA^+^)^28^, which are known to mediate rapid and substantial changes in cerebral blood flow^31^. In contrast, the responses of distal capillaries (diameter < 5 µm) to anesthesia are more variable, likely because only a subset of α-SMA-expressing pericytes on distal capillaries are contractile, thus regulating capillary diameter more locally^37,52,53^. Recent studies indicate that a segment of capillary can dilate following the targeted ablation of a single attached pericyte^54^. However, capillary pericytes may also indirectly affect the diameter of upstream arterioles through capillary-to-arteriole signaling, as demonstrated in previous research^30,34^.

This study has several limitations. First, using NG2-Cre mice to study vascular mural cells is less than ideal because the NG2 promotor in the cortex is expressed not only in pericytes and vSMCs but also in oligodendrocyte precursor cells and some scattered astrocytes^37,39,52,53,55–57^. To address this concern, we performed immunostaining for PDGFRβ^36^ and Kir6.1^26^ in the cortex of NG2-Cre mice. Our analyses showed that the majority of cortical NG2^+^ cells were positive for PDGFRβ and Kir6.1, confirming that our Ca^2+^ imaging and functional studies primarily targeted mural cells. Previous research indicates that PDGFRβ and Kir6.1 are predominantly expressed in pericytes^26^, suggesting that SEVO-induced vasodilation is likely mediated mainly by pericytes. However, because PDGFRβ and Kir6.1 are also present in vSMCs, albeit at lower levels^26^, we cannot completely exclude their contribution to SEVO-induced vasodilation. Additionally, our pharmacological studies on Kir6.1 cannot rule out potential off-target effects on other cell types. Future research using more precise functional and genetic approaches is needed to better delineate the roles of pericytes and Kir6.1 signaling in mediating the vasodilatory response to volatile anesthetics.

In summary, our studies demonstrated that volatile anesthetics (SEVO or ISO) rapidly and robustly induce dilation of cortical arterioles and arteriole-proximate capillaries in adult mice, a response mediated by vascular mural cells through Kir6.1 signaling. The diminished cerebral vasodilation observed in infant mice may be attributed to their lower density of pericyte-like mural cells and reduced Kir6.1 expression. Future research will investigate whether developmental differences in vascular mural cells, especially pericytes, and their associated vasodilatory responses to anesthesia play a role in anesthesia-induced developmental neurotoxicity.

## Methods

### Animals

We obtained C57BL/6J mice (stock no. 000664), NG2-Cre mice expressing Cre recombinase under the control of the mouse NG2 (*Cspg4*) promoter (029926), Ai162 mice expressing floxed GCaMP6s (031562), and Ai14 mice expressing floxed tdTomato (007914) from the Jackson Laboratory (Bar Harbor, ME, USA). Transgenic mice expressing GCaMP6s specifically in cortical pyramidal neurons, *Thy1*-GCaMP6s founder line 3^58^, were bred in-house and used for neuronal Ca^2+^ imaging. NG2-Cre;Ai162 and NG2-Cre;Ai14 mice were used for Ca^2+^ imaging and morphological studies of vascular mural cells, respectively. Mice were group-housed in temperature-controlled rooms with a 12-h light-dark cycle at the Columbia University animal facility (New York, NY, USA). We have complied with all relevant ethical regulations for animal use. All animal procedures were approved by the Institutional Animal Care and Use Committee (IACUC) at Columbia University, consistent with the National Institutes of Health (NIH) Guide for the Care and Use of Laboratory Animals.

### Exposure groups and anesthesia conditions

Mice of different ages (P13–14, P16–19, and P60–90), with each age group having an equal distribution of sexes and derived from at least two litters, were exposed to SEVO or ISO to achieve a surgical plane of anesthesia for up to 60 min. Awake mice with a cranial window implanted were head-fixed under a two-photon microscope and connected with a nose cone appropriately sized for each age group. Anesthesia was induced and maintained with SEVO 2.5% or ISO 1.5% in air. The induction phase took approximately 5 min until the response to a toe pinch was lost. Respiratory rate and skin color were monitored visually, and a heating pad maintained the animals’ body temperature at ∼37 °C. After 1 h of exposure, SEVO or ISO administration was discontinued, and the animals were euthanized for IHC studies. In some experiments, animals were allowed to recover for up to 90 min after SEVO discontinuation to monitor changes in vessel diameter during the recovery phase. Additionally, separate sets of animals from all age groups were exposed to SEVO for 15 min under similar conditions to measure arterial blood gases at this time point during anesthesia.

### Physiological measurements

Animals were exposed to SEVO 2.5% as described above. Respiratory rates were determined by counting thoracic breathing movements over a 30-s period and calculated as breaths per minute. Subsequently, approximately 0.1 ml of blood was withdrawn from the carotid artery and immediately transferred to a CG8^+^ cartridge for blood gas analysis using an iSTAT analyzer (Abbott Laboratories, Illinois, USA).

### Cranial window implantation

The surgical procedure for cranial window preparation has been described previously^59,60^. In brief, mice were anesthetized with an intraperitoneal (i.p.) injection of 100 mg kg^−^^1^ ketamine and 15 mg kg^−1^ xylazine (Covetrus, Portland, ME, USA). A head frame (18.5 × 11.5 mm; ~0.5 g; CF-10; Narishige, Amityville, NY, USA) was secured to the animal’s skull using glue (LOCTITE® 495, R.S. Hughes Co., Sunnyvale, CA, USA) and dental cement (Metabond, Parkell, Edgewood, NY, USA). A thinned-skull window of 1.5–2.0 mm diameter was created at coordinates anterior-posterior (AP) −1.0 mm and medial-lateral (ML) 1.2 mm. A glass coverslip was then carefully affixed over the thinned skull to protect the area. Upon awakening, mice were returned to their home cages. Imaging of cortical vessels was conducted on the following day.

### Electrode implantation and recording

Following the attachment of a head frame, a custom-designed headstage (weight ~0.12 g) for EEG and EMG recording was implanted while the animal was under anesthesia. The headstage included two EEG electrodes (perfluoroalkoxy alkane-coated silver wire; ø 0.0055-inch; A-M Systems, Sequim, WA, USA) and two EMG electrodes (epoxy-coated copper wire; ø 0.10 mm). These electrodes were soldered to a female miniature multichannel connector (4.5 × 2.5 mm) and insulated with a seal coat of quick-cure epoxy resin (G14250; ThorLabs, Newton, NJ, USA). The EEG electrodes were positioned near the lambda and midline, spaced 500 μm apart, and inserted through the skull to make contact with the dura mater. They were secured in place using glue and dental cement. The EMG electrodes were placed in the nuchal muscles. The headstage was then affixed to the rear beam of the head frame using dental cement. During recording, the connector was linked to a preamplifier, which was connected to a multichannel amplifier. Signals were amplified 500 times with a sampling frequency of 5 kHz.

### Vessel labeling

To label arteries, mice received an i.v. injection via the tail vein of Alexa Fluor 633 Hydrazide (2 mg kg^−1^; A30634; Thermo Fisher, Waltham, MA, USA), a fluorescent dye specific to arteries^27^. In some experiments, mice were injected with 70 kDa Rhodamine B isothiocyanate–Dextran (1 mg in 100 µl PBS; R9379; Sigma-Aldrich, St. Louis, MO, USA) immediately before imaging to label the vasculature. To measure vessel diameter, a perpendicular line was drawn across the vessel, and the distance between the vascular walls, visualized by fluorescent dyes or light-field imaging, was measured to determine the diameter (Fig. 1c–e).

### In vivo two-photon imaging

In vivo imaging was conducted using a Scientifica two-photon system (Uckfield, East Sussex, UK) equipped with a Ti:Sapphire laser (Vision S, Coherent, Santa Clara, CA, USA). The laser was tuned to 810 nm for imaging arterioles and 920 nm for imaging Ca^2+^ activity, vasculature, and NG2^+^ cell morphology. All experiments used a 20× (1.00 N.A.) or 25× (1.05 N.A.) objective immersed in artificial cerebrospinal fluid (ACSF), with a digital zoom of 1× for large arterioles and 2× for small arterioles and capillaries. Imaging was conducted at a frame rate of 1.69 Hz (2-µs pixel dwell time) with resolutions of 512 × 512 pixels for time-lapse imaging and 1024 × 1024 pixels for Z-stack imaging. To minimize potential phytotoxicity, both imaging duration and repetitions were kept to a minimum. Image acquisition was controlled using ScanImage software (Vidrio Technologies, Ashburn, VA, USA). Z-stack images of the vasculature were collected to detect arterioles and venules with diameters ranging from 2 to 45 μm within 400 µm below the pial surface. Vessel segment diameters were measured using NIH ImageJ software. For Ca^2+^ imaging in NG2^+^ mural cells, GCaMP^+^ cells (identified by their bump-like soma) in contact with vasculature labeled by Rhodamine (see ‘Vessel labeling’) were presumed to be mural cells. Changes in their fluorescence intensity following anesthetic exposure were normalized to the baseline fluorescence measured under awake conditions. For Ca^2+^ imaging in cortical pyramidal neurons, time-lapse images were captured at depths of 200–350 μm below the pial surface to detect pyramidal somas in layers 2/3^61^. Imaging data were analyzed using CaImAn (Python version 1.9.12; available on github.com) to report Δ*F/F*_0_ (that is, *F*–*F*_0_/*F*_0_ × 100%, where *F*_0_ is the baseline fluorescence) as an indicator of neuronal Ca^2+^ activity.

### Immunohistochemistry and confocal imaging

Mice were transcardially perfused with phosphate-buffered saline (PBS) followed by 4% paraformaldehyde (PFA) in PBS. Brains were post-fixed in 4% PFA for 3 days and coronally sectioned at 50-μm thickness using a Leica vibratome (VT 1000 S, Buffalo Grove, IL, USA) for IHC. In some experiments (Fig. 4a), the cortical surface was transversely sectioned at 50-μm thickness to visualize pial arterioles. Sections were further post-fixed in 4% PFA for 1 h and rinsed three times with PBS. Floating sections were permeabilized and blocked in 0.1% Triton X-100 with 5% goat or donkey serum in PBS for 1.5 h at room temperature. Sections were then incubated overnight at 4 °C with one or more of the following primary antibodies: rat anti-NG2-Alexa Fluor 488 conjugated (1:300; AB5320A4; Sigma-Aldrich), mouse anti-α-SMA Cy3 conjugated (1:200; C6198; Sigma-Aldrich), goat anti-PDGFRβ (1:200; AF1042; R&D Systems, Minneapolis, MN, USA), rabbit anti-laminin (1:300; ab11575; Abcam, Cambridge, MA, USA), rabbit anti-Kir6.1 (KCNJ8) (1:200; APC-105; Alomone, Jerusalem, Israel), and rabbit anti-cleaved caspase-3 (1:300; 9661; Cell Signaling, Danvers, MA, USA). For immunostaining with the Kir6.1 antibody, epitope retrieval was performed by boiling sections in PBS for 15 min before incubation with primary antibodies. After three washes with PBS, sections were incubated for 2 h at room temperature with fluorophore-conjugated secondary antibodies: goat anti-rabbit Alexa Fluor 488 (A11008; Invitrogen, Waltham, MA, USA), goat anti-mouse Alexa Fluor 488 (A11029; Invitrogen), goat anti-mouse CF543 (20299; Biotium, Fremont, CA, USA), goat anti-mouse CF647 (20299; Biotium), goat anti-rabbit Alexa Fluor 660 (A21074; Invitrogen), goat anti-mouse Alexa Fluor 660 (A21055; Invitrogen), donkey anti-mouse Alexa Fluor 546 (A10036; Invitrogen), donkey anti-rabbit Alexa Fluor 488 (A21206; Invitrogen), donkey anti-rabbit CF488A (20015; Biotium), donkey anti-mouse CF543 (20305; Biotium), and donkey anti-goat CF647 (20048; Biotium). Following three PBS washes, sections were mounted with (010020; SouthernBiotech, Birmingham, AL, USA) or without DAPI (010001; SouthernBiotech) for confocal imaging.

Confocal imaging was conducted using a Nikon Ti laser scanning confocal system (Melville, NY, USA) equipped with a 10× or 20× objective. Images were acquired at a resolution of 1024 × 1024 pixels with a pixel size of 0.622 μm. Z-stacks images were obtained with 2-μm step sizes, and maximum intensity projections were generated to create final multichannel images, which were analyzed using ImageJ software. To quantify NG2^+^ pericyte density in the cortex, cells displaying pericyte morphology and colocalizing with DAPI-labeled nuclei were counted. Cell density was calculated as the number of cells per mm^2^ of cortical area.

### Optogenetic manipulation of NG2^+^ cells

To optogenetically manipulate NG2^+^ cells, 0.2 µl of AAV encoding Cre-dependent halorhodopsins (NpHR; AAV9-EF1a-DIO-eNpHR3.0-EYFP; titer 3.9 × 10^13^ genome copies ml^−1^; 26966; Addgene, Watertown, MA, USA) or channelrhodopsins (ChR2-SSFO; AAV1-Ef1a-DIO-hChR2(C128S/D156A)-EYFP; titer 1.0 × 10^13^ gc ml^−1^; 35503; Addgene) or control vectors (EYFP; AAV1-Ef1a-DIO-EYFP; titer 1.0 × 10^13^ gc ml^−1^; 27056; Addgene) were injected into the cortex of NG2-Cre mice at the coordinates: AP −1.2 mm, ML 1.5 mm, and subpial (SP) 150 µm. Experiments were conducted 2 weeks after viral injection.

To silence NG2^+^ cells by inducing membrane hyperpolarization, continuous yellow light (595 nm; ~5 mW mm^−^^2^ in power) was directed onto the cortical surface for 600 s to activate eNpHR3.0 expressed in NG2^+^ cells, thereby triggering Cl^−^ currents in these cells. For persistent activation of NG2^+^ cells by inducing membrane depolarization, a stabilized step-function channelrhodopsin variant, ChR2-SSFO, was expressed in NG2^+^ cells. A brief pulse of blue light can keep ChR2-SSFO open for more than 30 min, allowing cation influx and cell depolarization, with immediate closure upon exposure to yellow light. To depolarize NG2^+^ cells, a 2-s flash of blue light (470 nm; ~5 mW mm^−2^ in power) was delivered to the cortical surface of mice, with or without SEVO exposure. After 10 min, a 2-s flash of yellow light (595 nm; ~5 mW mm^−2^ in power) was used to close the channels and reverse depolarization. Changes in vessel diameter in response to optogenetic stimulation of NG2^+^ cells were measured using two-photon imaging.

### Pharmacological manipulation of Kir6.1

To locally activate or inhibit Kir6.1, 0.1 μl of pinacidil (50 μM; P154; Sigma-Aldrich) or glibenclamide (10 μM; 0911; Tocris), dissolved in ACSF, was stereotaxically injected into the targeted cortical region (coordinates: AP −1.2 mm, ML 1.5 mm, SP 200 µm). The injection was performed using a picospritzer (5 p.s.i., 12 ms pulse width, 0.3–0.4 Hz) at a rate of ~10–15 min per 0.1 μl.

### Local ablation of NG2^+^ cells

To locally ablate NG2^+^ cells, adult NG2-Cre mice were injected with 0.2 μl of AAV encoding Cre-dependent diphtheria toxin receptor (DTR) (AAV2-FLEx-DTR-GFP; titer 1.3 × 10^13^ gc ml^−1^; 124364; Addgene) into the right cortex, and a control reporter (AAV2-FLEx-GFP; titer 1.2 × 10^13^ gc ml^−1^; 51502; Addgene) into the left cortex, at the coordinates described above. Two weeks post-injection, mice received a single i.p. injection of 100 ng g^−1^ diphtheria toxin (DT) (D0564; Sigma-Aldrich) to ablate NG2^+^ cells expressing DTR (day −7). On day −2, mice underwent surgical preparation for head frame mounting. Two-photon imaging was conducted on day 0, as described above. Following imaging, mice were sacrificed for IHC assessment of NG2^+^ cell ablation.

### Statistics and reproducibility

Statistical analyses were conducted using Prism software, version 8.0 (GraphPad; San Diego, CA, USA). Data are presented as mean ± SEM (standard error of the mean) or SD (standard deviation of the mean), as specified. The time course of vasodilation, cytosolic Ca^2+^ concentrations (or activity) were compared using a two-tailed paired Student’s *t*-test. Differences between two populations were assessed using a two-tailed unpaired Student’s *t*-test, Mann-Whitney *U* test, Kolmogorov-Smirnov test, or two-way ANOVA, as indicated in the figure legends. No data points were excluded from the analysis, and variance was similar between compared groups. Statistical significance was defined as *P* < 0.05.

## Supplementary information

Supplementary information

Description of Additional Supplementary File

supplementary data

## Data Availability

All data are available in the main text or the supplementary materials. Source data underlying plots are provided as Supplementary Data.
