# Supplementary material for: Age-dependent cerebral vasodilation induced by volatile anesthetics is mediated by NG2+ vascular mural cells
Source: Commun Biol. 2024 Nov 15;7:1519. doi: 10.1038/s42003-024-07200-7 (PMC11568297; doi:10.1038/s42003-024-07200-7)
Supplement: Supplementary file 1 — Supplementary information [file 42003_2024_7200_MOESM1_ESM.pdf]

Age-dependent cerebral vasodilation induced by volatile anesthetics is mediated by NG2$^+$ vascular mural cells

Hang Zhou[1, 2, 3], Viola Neudecker[1, 3], Jose F. Perez-Zoghbi[1, 3],
Ansgar M. Brambrink[1*], Guang Yang[1*]

[1] Department of Anesthesiology, Columbia University Irving Medical Center, New York, NY, 100032, USA

[2] Present address: Faculty of Life and Health Sciences, Shenzhen University of Advanced Technology (SUAT), Shenzhen, Guangdong Province, 518107, China

[3] These authors contributed equally.

***Corresponding Author**
Guang Yang
Department of Anesthesiology, Columbia University Irving Medical Center, New York, NY, 10032, USA
E-mail: gy2268@cumc.columbia.edu

Ansgar M. Brambrink
Department of Anesthesiology, Columbia University Irving Medical Center, New York, NY, 10032, USA
E-mail: amb2457@cumc.columbia.edu

	**Adult**		**P18**		**P14**	
**Parameter**	**mean**	SEM	**mean**	SEM	**mean**	SEM
**pH**	**7.27**	0.02	**7.19**	0.02	**7.28**	0.01
**pCO_2 (mmHg)**	**40.9**	1.6	**57.5**	1.1	**64.7**	1.9
**pO_2 (mmHg)**	**106.0**	1.9	**135.4**	6.8	**101.6**	5.0
**BE (mmol L^{-1})**	**-7.8**	1.4	**-6.5**	1.1	**3.3**	0.8
**HCO_3^- (mmol L^{-1})**	**19.1**	1.1	**21.8**	0.7	**30.1**	0.6
**sO_2 %**	**97.6**	1.3	**98.1**	0.8	**95.9**	0.7
**Na^+ (mmol L^{-1})**	**143.2**	0.2	**138.7**	0.4	**136.6**	0.9
**K^+ (mmol L^{-1})**	**3.9**	0.8	**3.2**	0.3	**3.9**	0.6
**iCa^{2+} (mmol L^{-1})**	**1.2**	0.1	**1.4**	0.2	**1.4**	0.0
**Glu (mg dL^{-1})**	**248.0**	0.0	**159.4**	0.0	**122.3**	0.0
**Hct**	**41.4**	11.1	**22.9**	3.8	**27.0**	4.1
**Hb (g dL^{-1})**	**14.1**	1.6	**7.8**	0.7	**9.2**	1.0
**Resp. Rate (min^{-1})**	**134**	0.5	**66**	0.2	**85**	0.3

Supplementary Table 1 | Arterial blood gas parameters in different age groups after 15 min SEVO exposure.
BE (base excess), Hct (hematocrit), Hb (hemoglobin). Data are mean ± SEM from $n = 5$ (3 female, 2 male) for adult, $n = 7$ (4 female, 3 male) for P18, and $n = 10$ (6 female, 4 male) for P14 mice.

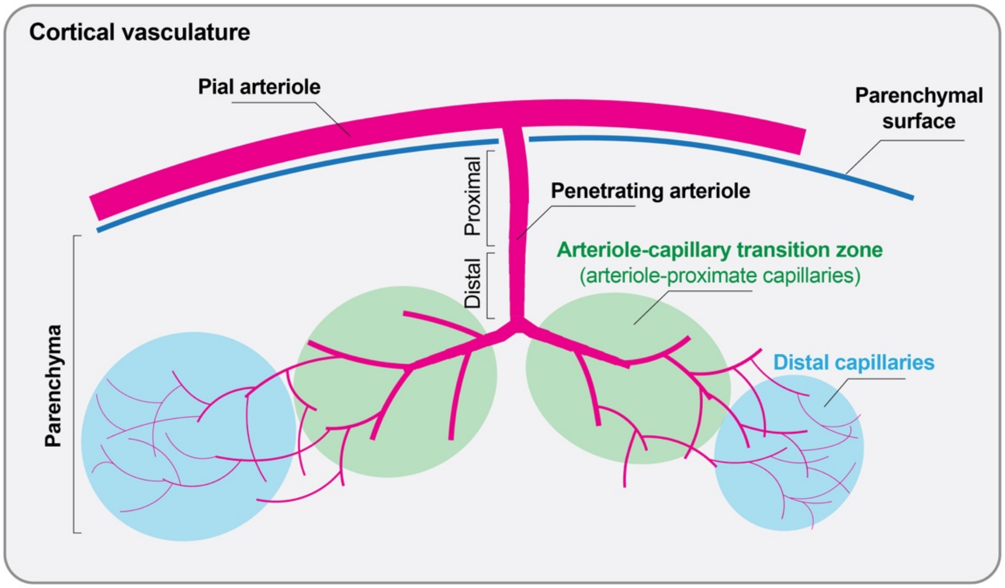

Supplementary Figure 1 | Terminology of cortical vasculature.

Diagram illustrating the terms used to define different segments of cortical vasculature.

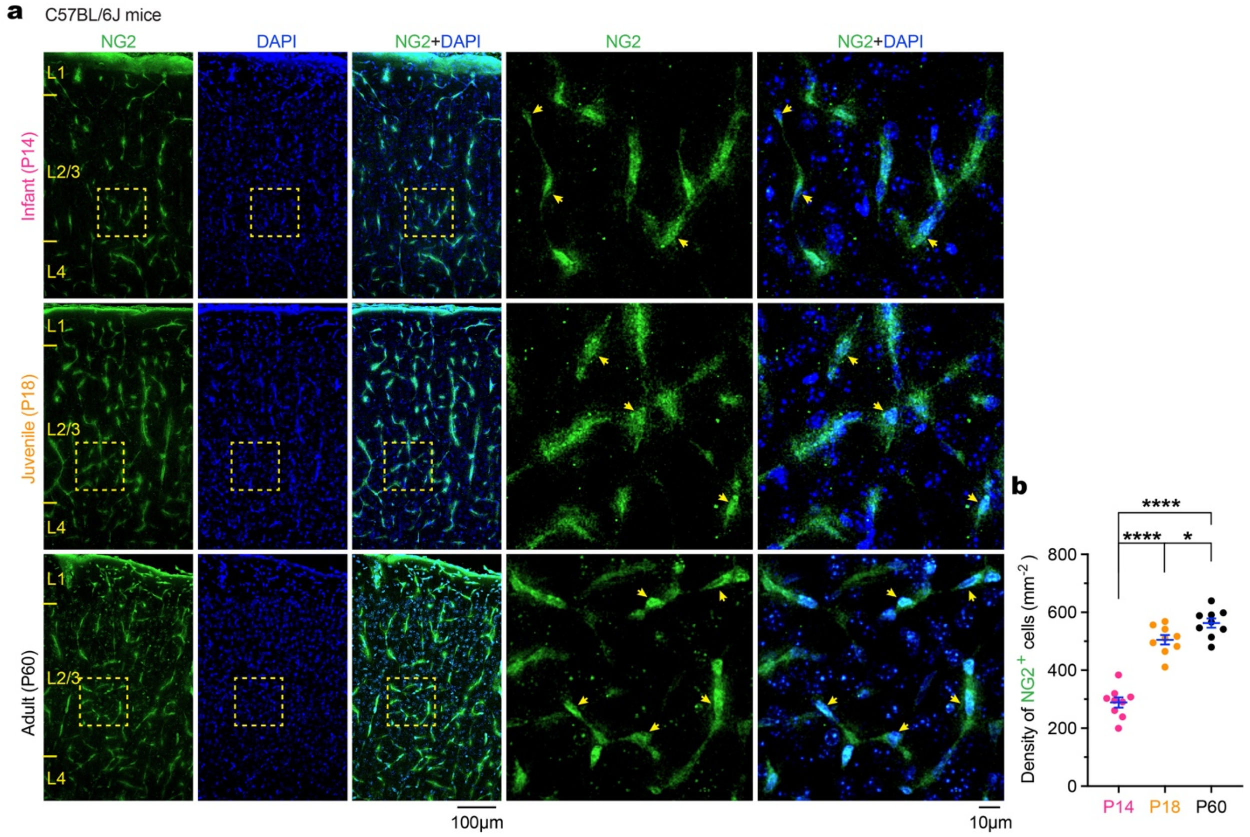

Supplementary Figure 2 | Immunohistochemistry analysis of NG2$^+$ cells in the mouse cortex at different ages.

a, Immunofluorescence images of the mouse cortex stained for NG2 (green) and DAPI (blue). Boxed regions are shown at higher magnification on the right. Arrows indicate NG2$^+$ cells with pericyte morphology. **b**, Density of NG2$^+$ cells with pericyte morphology in the cortex of P14, P16, and P60 mice (n = 9 slices from three mice per group; P = 0.0336, and < 0.0001 for else). P14, 288.8 ± 17.4; P18, 505.1 ± 16.3; P60, 562.8 ± 16.1. Data are mean ± SEM. *P < 0.05, ****P < 0.0001; *NS*, not significant; by two-tailed Mann-Whitney U tests.

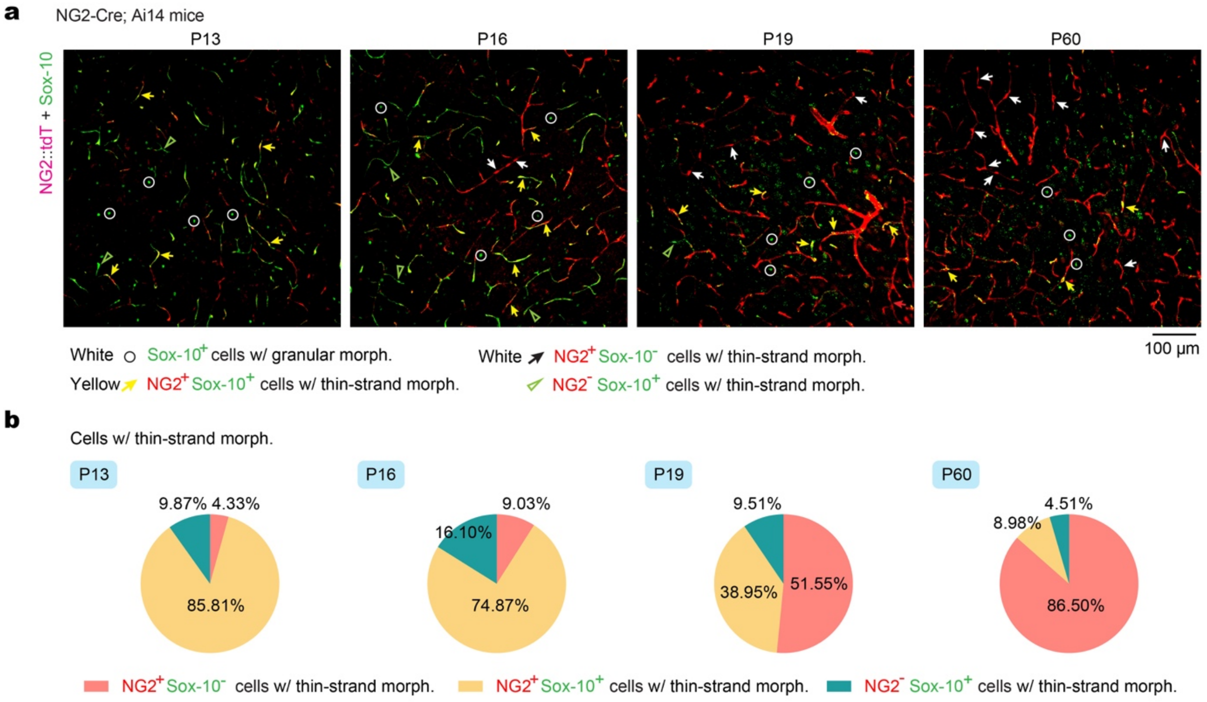

Supplementary Figure 3 | Analysis of Sox-10⁺ oligodendrocyte precursor cells and NG2⁺ cells in the mouse cortex at different ages.

a, Confocal images showing cortical NG2$^+$ cells expressing tdTomato (tdT) and oligodendrocyte precursor cells (OPCs) stained for Sox-10 in mice at various ages. Circles, arrows and arrowheads indicate cells with thin-strand or granular morphology, with or without NG2 and Sox-10 expression, as depicted below the images. **b**, Percentage of cortical cells exhibiting thin-strand morphology and labeled with NG2 and/or Sox-10 at various ages.

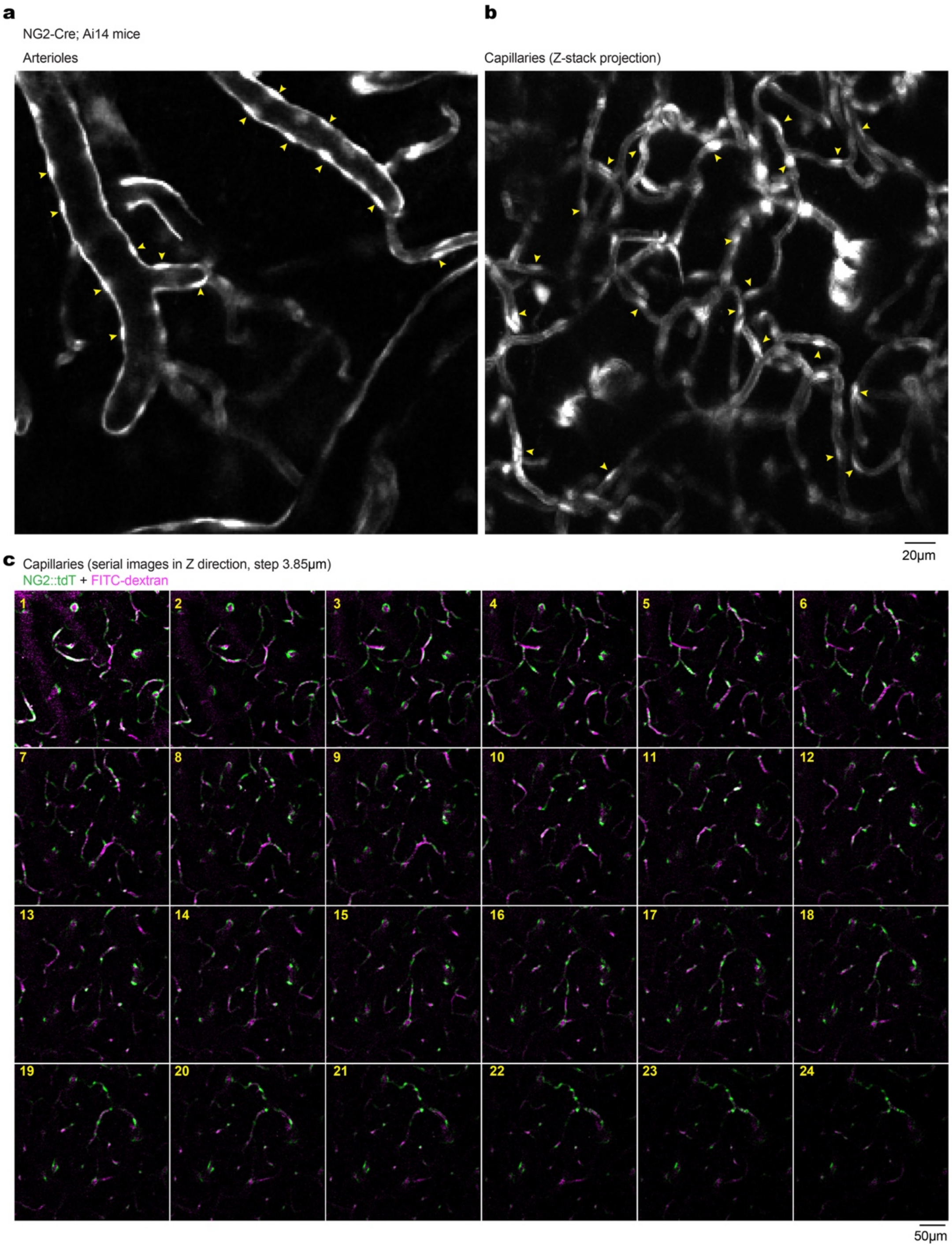

Supplementary Figure 4 | NG2^{+} cells are closely associated with vasculature in the mouse cortex.

a, Representative two-photon image showing tdTomato-expressing NG2^{+} cells in close proximity to pial blood vessels in the cortex of NG2-Cre;Ai14 mice. Arrows indicate NG2^{+} cell somas. **b**, Maximum intensity projection of two-photon Z-stack images collected at the subpial depth of 100–192 µm, showing the colocalization of NG2^{+} cells and capillaries in the cortex of NG2-Cre;Ai14 mice. Arrows indicate NG2^{+} cell somas. **c**, Individual frames of the two-photon Z-stack displayed in (b), with blood flow visualized by i.v. injection of FITC-dextran. Z-step size was 3.85 µm.

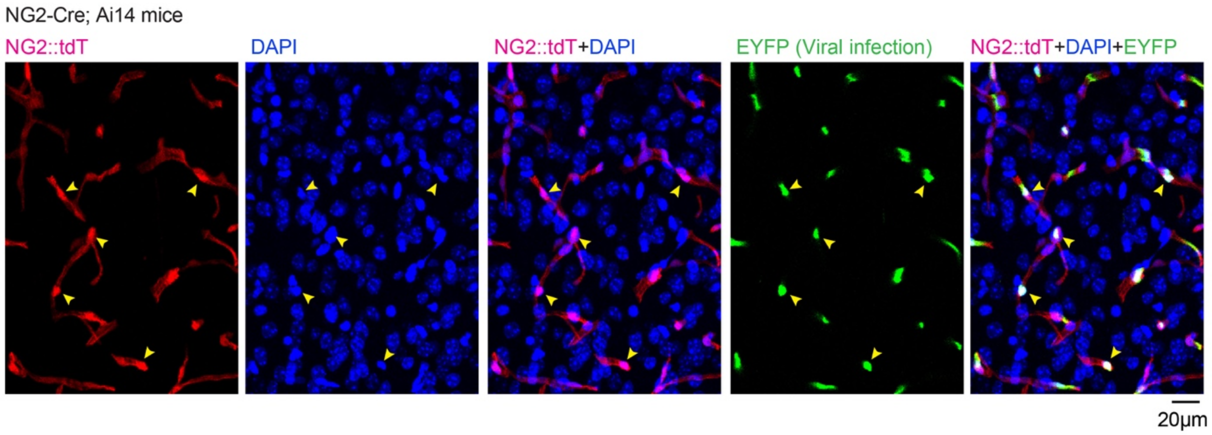

Supplementary Figure 5 | Representative images of NG2$^+$ cells with pericyte morphology.

Immunofluorescence images showing tdTomato (tdT)-labeled NG2$^+$ cells, DAPI-stained nuclei, and virally expressed EYFP (AAV-ChR2-SSFO-EYFP) in the cortex of NG2-Cre;Ai14 mice. Note that the virally transduced EYFP is primarily expressed in the somatic region of NG2$^+$ cells.
